# Case report: Successful treatment of human diabetic foot ulcer using low-intensity diagnostic ultrasound combined with microbubbles: Two cases

**DOI:** 10.3389/fendo.2022.1046896

**Published:** 2022-11-25

**Authors:** Xiaojuan Zhang, Ying Cheng, Ling Pei, Jie Tao, Rui Wang, Zhong Chen

**Affiliations:** ^1^ Department of Ultrasound, the General Hospital of Western Theater Command, Chengdu, China; ^2^ Department of Endocrinology, the General Hospital of Western Theater Command, Chengdu, China

**Keywords:** diabetic foot ulcer, diagnostic ultrasound, microbubble, ultrasound treatment, case report

## Abstract

**Background:**

Diabetic foot ulcer (DFU) is one of the serious complications of diabetes, which has high disability rate and mortality. Low-intensity ultrasound combined with microbubbles in blood circulation can enhance the blood perfusion effect of local soft tissue, which has the potential to promote the healing of diabetic ulcer. Here, we report how this method was used to help the healing of two patients with chronic refractory DFUs.

**Case Presentation:**

In case 1, a 56-year-old man with 3-years history of type 2 diabetes had a 3.0×2.0 cm ulcer which infected with staphylococcus aureus on his right calf for more than half a month. In case 2, a 70-year-old man with 10-years history of type 2 diabetes presented with an 8-month right heel ulcer that developed to 7.5×4.6 cm. And he also had hyperlipidemia, hypertension, and renal impairment. Both patients were enrolled in our study to receive treatment of low-intensity diagnostic ultrasound (LIDUS) combined with microbubbles. They were discharged after a 20-minute daily standard treatment for 7 consecutive days. The ulcers in both cases completely healed in 60 days and 150 days, respectively, and haven’t recurred for more than one year of follow-up.

**Conclusion:**

It is feasible, safe, and effective to use commercial LIDUS combined with commercial microbubbles in the treatment of diabetic lower extremity ulcers. This study may provide an innovative and non-invasive method for the treatment of DFUs.

## Introduction

Diabetic foot ulcer, as one of the serious complications of diabetes, has brought heavy economic and public health burden to the society due to its high incidence (15-25%) (1), high disability rate and high mortality (2) in diabetic patients.

Microcirculatory dysfunction is an important cause of DFU. On one side, hyperglycemia and hyperinsulinemia promote characteristic extensive endothelial hyperplasia, basement membrane thickening, and even calcification in arterioles, leading to ischemia-hypoxia and poor perfusion in foot soft tissue (3). On the other, hyperglycemia and oxidative stress lead to endothelial dysfunction, characterized by impaired auto-regulation of micro vessels and a blunted response to vasodilatory stimuli, thereby exacerbating functional perfusion defects in the limbs (4).

Endovascular shear force is an important means to regulate endothelium-derived vasoactive substances and control micro vasodilation (5). According to this, a series of related drugs and modified endogenous active substances have been developed to treat tissue ischemia through improving microcirculation perfusion.

Low-intensity pulse ultrasound, is a kind of ultrasonic energy mainly with mechanical effect, but not thermal effect. The shear force, micro jet and shock wave generated by ultrasonic pulse produce a series of physical and biological effects, which are widely used in the therapeutic field. Perfusion effect is one of these effects, which is to enhance local blood perfusion in tissues by setting appropriate acoustic parameters (6). The microbubbles in the circulation, as cavitation nuclei, could make the ultrasound produce very high shear force and multiply the effect of local blood flow enhancement. Therefore, when low-intensity ultrasound is combined with microbubbles, it has a very good potential for the treatment of tissue ischemic diseases.

In this report, we presented two complete healing cases of refractory DFU treated by commercial LIDUS combined with commercial microbubbles for the first time.

## Materials and methods

A GE LOGIQ 9 ultrasound scanner (GE Healthcare, Waukesha, WI) equipped with a 9L Linear array probe (GE Healthcare) was used for both conventional ultrasonography and Contrast-Enhanced Ultrasonography (CEUS). In conventional ultrasonography, thyroid imaging mode was used with a frequency of 9MHz and an imaging depth of 4cm. In CEUS, “Contrast” key was clicked.

An Acuson S2000 ultrasound scanner (SIMENS Healthcare, Erlangen, Germany) equipped with a 9L4 Linear array probe was used for all treatments. Contrast pulse sequencing (CPS) mode were used to monitor microbubble perfusion and an intermittent flash of high MI impulses. The frequency of flash was set at 4 MHz, Imaging depth at 4 cm, with a frame rate of 50 frames per second and an MI of 0.86 (79% acoustic output power).

Same CEUS imaging sections of the ulcerative and surrounding soft tissue before and after treatment were used for perfusion evaluation, chartered with adjacent vessels or bony structures. All parameters of ultrasound were consistent in both patients during diagnosis and treatment.

The microbubbles used for ultrasonic diagnosis and treatment were SonoVue (Bracco Imaging Scandinavia AB, Oslo, Norway), a commercial ultrasound contrast agent. The microbubble suspension with a concentration of 11.8 mg/mL were prepared according to the manufacturer’s instructions, with 59 mg sulfur hexafluoride lyophilized powder mixed with 5 mL of normal saline. A 140 ×110 ×7 mm acoustic coupling pad (Foshan SiEn Technology Co., LTD., Guangzhou, China) was used during imaging and treatment procedures for better coupling.

After routine clinical debridement of the wound, the acoustic coupling pad was placed on the ulcerated skin area. CEUS was first performed on local tissue, and 2.4 ml microbubble suspension was injected rapidly through the cubital vein, followed by flushing with 5 ml normal saline. CPS angiography combined with microbubble Flash mode was used for treatment: “Microbubble Flash → Microbubble Contrast → Microbubble Flash → Microbubble Contrast”. In the first 5 minutes, the remaining circulating microbubbles from previous CEUS were used to mediate ultrasound therapy. Then another 5 ml of the prepared microbubble suspension was taken and injected slowly and continuously through the vein for 10 min. Finally, the remaining circulating microbubbles were used again to mediate ultrasound treatment for 5 min, and the total time of ultrasound treatment was 20 min. The ultrasound treatment cycle was 7 days, once a day, and the treatment process was the same for each time. Follow-up observation was conducted for 6 months.

## Case presentation

### Case 1

A 56-year-old man with 3-years history of type 2 diabetes fell to the ground while cycling two weeks ago, resulting in a skin ulceration on his right calf. He received basic debridement and daily dressing change at local hospital but the ulcer did not heal. Subsequently, the patient was admitted to the department of Endocrinology in our hospital. The patient was not taking any medication at admission.

**Figure 1:**
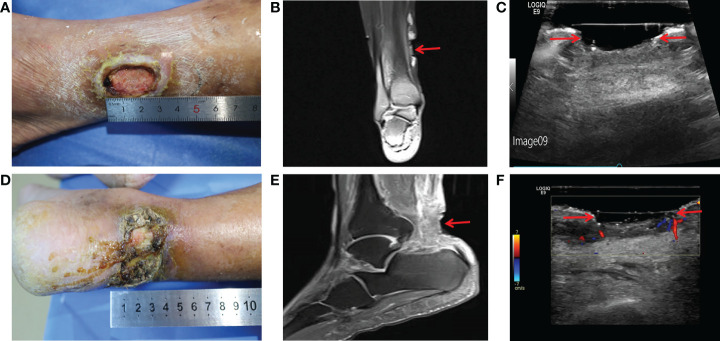
Imaging diagnosis of diabetic lower extremity ulcers before treatment for case 1 **(A-C)** and case 2 **(D-F)**. **(A)** Picture showed an ulcerated surface on the inner skin of the right calf, about 3.0×2.0 cm in size and 6 mm in depth; **(B)** MRI showed a local subcutaneous soft tissue defect at the medial margin of the right calf, with swelling in the margin and adjacent soft tissue space; **(C)** Gray-scale sonography showed a heterogeneous low echo area in the subcutaneous soft tissue of the medial side of the right calf; **(D)** Picture showed an ulcerated surface in the skin of the right heel, about 7.5 × 4.6 cm in size and 4 mm in depth, with necrosis and exudation; **(E)** MRI showed extensive swelling of the soft tissue and fascia in the lower part of the right calf with unclear and disordered layers; **(F)** CDFI showed local skin defects and discontinuity in the skin of the right heel. The blood flow signal in the low echo surface was not obvious, and a little blood flow signal could be seen in the periphery. The red arrows indicate the ulcer defects.

Physical examination showed an ulcerated surface on the patient’s medial right calf, about 3.0 ×2.0 cm in size, with a depth of about 6 mm (**Figure 1A**). The granulation tissue was relatively fresh, with a little dark red bloody exudate on the surface, and the surrounding soft tissues were red, swollen and slightly tender. Laboratory examination showed increased fasting blood glucose (16.2 mmol/L), increased HbA1c (7.10%), normal liver and renal function indexes. Secretion culture from the ulcer indicated an infection of Staphylococcus epidermidis (**Table 1**). Magnetic resonance imaging (MRI) examination showed that the ulcer did not involve bone tissue (**Figure 1B**). Color Doppler Flow Imaging (CDFI) showed no significant abnormalities in peripheral arteries (**Figure 1C**). Ankle-brachial index (ABI) was normal (ABI=1.20), Current perception threshold (CPT) was 0.00, and there was no abnormal sensation (**Table 1**). Based on these evidence, the patient preliminary diagnosed as diabetic foot ulcer (Grade 3 of Wagner classification).

**Table 1:** Details of blood routine, blood glucose level and other testing.

Indices	Pre-therapy	Post-therapy	Normal range
Case 1			
Total bilirubin(TBIL)	5.8 umol/L	5.6 umol/L	5-28 umol/L
Aspartate aminotransferase(AST)	13.1 IU/L	14.6 IU/L	9-60 IU/L
Alanine aminotransferase(ALT)	18.9 IU/L	22.1 IU/L	15-45 IU/L
Urea	6.95 mmol/L	5.3 mmol/L	2.9-7.2 mmol/L
Serum creatinine(SCr)	71 umol/L	70 umol/L	44-133 umol/L
Uric Acid(UA)	302 umol/L	328 umol/L	100-432 umol/L
Glucose(GLU)	16.29 mmol/L	6.3 mmol/L	3.80-6.10 mmol/L
Glycosylated hemoglobin(HbA1c)	7.10%	6.9%	4.00-6.50%
Ankle-brachial index (ABI)	1.2	0.96	0.9-1.3
Current perception threshold (CPT)	0.00	7.00	0.00
Case 2			
Total bilirubin(TBIL)	11.3 umol/L	12.6 umol/L	5-28 umol/L
Aspartate aminotransferase(AST)	22.9 IU/L	36.3 IU/L	9-60 IU/L
Alanine aminotransferase(ALT)	22.5 IU/L	22.6 IU/L	15-45 IU/L
Urea	16.7 mmol/L	33.14 mmol/L	2.9-7.2 mmol/L
Serum creatinine(SCr)	261 umol/L	292 umol/L	44-133 umol/L
Uric Acid(UA)	559 umol/L	575 umol/L	100-432 umol/L
Creatinine Clearance(CCR)	33.7 mL/min	29.6 mL/min	>80 mL/min
Glucose(GLU)	5.91	10.77	3.80-6.10 mmol/L
Glycosylated hemoglobin(HbA1c)	8.90%	–	4.00-6.50%
Ankle-brachial index (ABI)	1.12	1.32	0.9-1.3
Current perception threshold (CPT)	8.37	8.37	0.00

**Figure 2:**
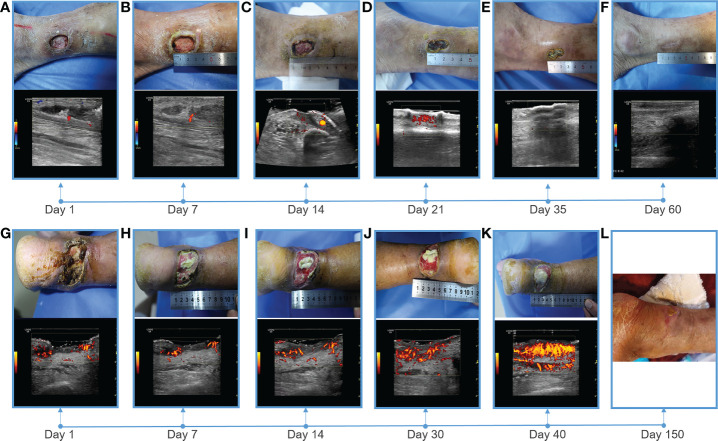
Showing of skin wounds and CDFI or PDI ((Power Doppler Imaging)) ultrasound imaging for progress in the treatment of diabetic lower extremity ulcers in two cases. Wound conditions at the **(A)** 1th, **(B)** 7th, **(C)** 14th, **(D)** 21th, **(E)** 35th, and **(F)** 60th day of post therapy showed the gradual healing of ulceration for case 1; Wound conditions at the **(G)** 1th, **(H)** 7th, **(I)** 14th, **(J)** 30th, **(K)** 40th, and **(L)** 150th day of post therapy showed the gradual healing of ulceration for case 2. PDI showed that the blood flow of the soft tissue around the ulcer gradually increased, and the blood flow was very abundant before healing.

**Figure 3:**
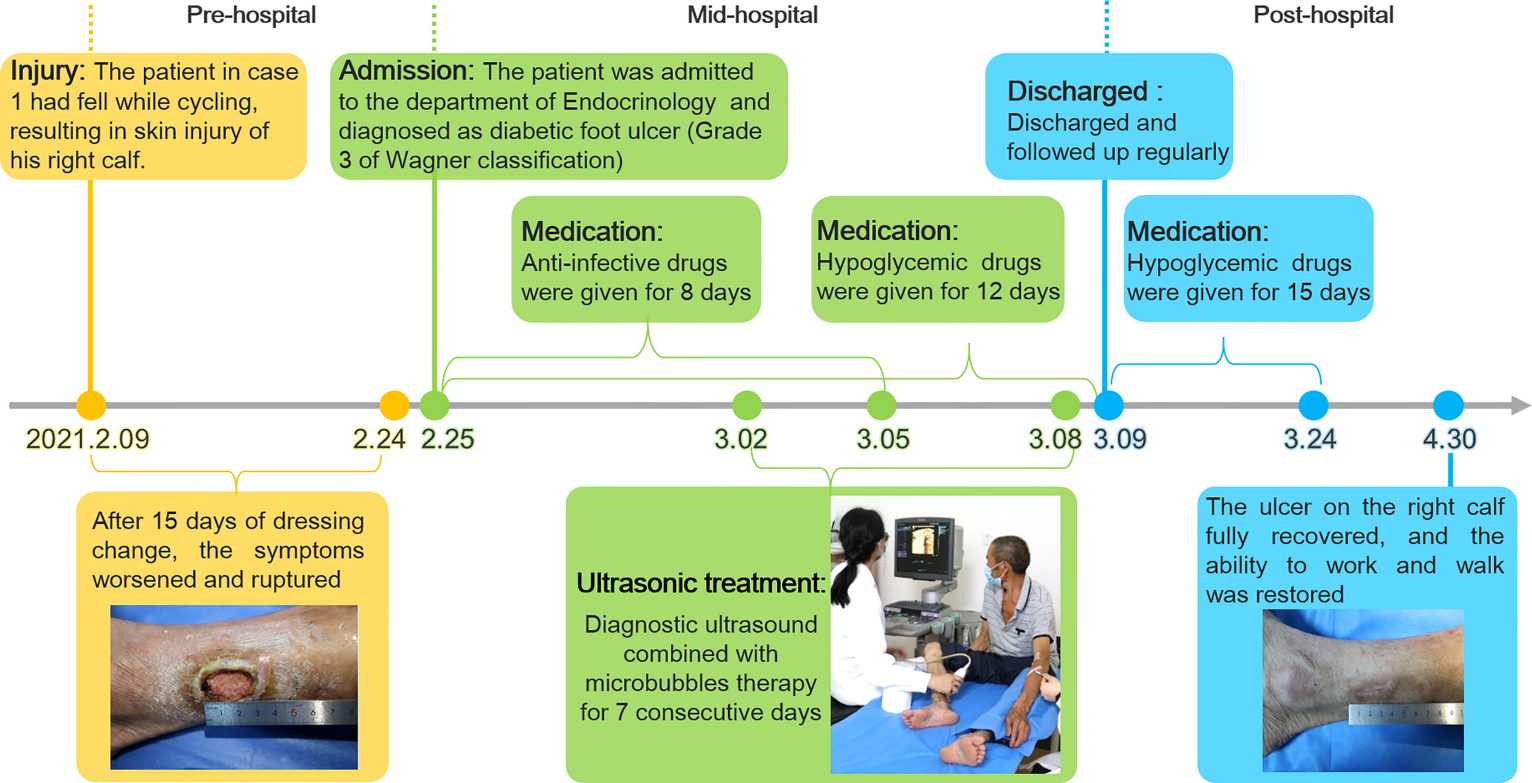
The timeline of the treatment process of case 1 from the day of injury to the day of healing.

After admission, the patient received antibiotic therapy for 8 days (intravenous cefotiam hydrochloride, 1g/8h, once a day). The ulcer wound dressing were changed once a day. For blood glucose control, the patient also received subcutaneous injection of recombinant human insulin (4 IU, three times a day), before each meal, subcutaneous injection of protamine human insulin (10 IU, once a day), at 22:00 every day, oral metformin hydrochloride sustained-release tablet (0.5 g, twice a day), oral sitagliptin phosphate tablet (100 mg, once a day). LIDUS combined with microbubbles therapy was performed once a day from the 5th day of admission (**Figure 2A**). On the 8th day of anti-infection, there was no purulent secretion in the wound, and the redness and swelling of the surrounding block were alleviated. On the 12th day of blood glucose control, the blood glucose level reduced to 6.3 mmol/L (**Table 1**). On the 12th day after enrolling in the LIDUS therapy, the ulcer area also decreased from 3.0 ×2.0 cm to 2.8 × 1.4 cm and its depth decreased from 6 mm to 4 mm, filled with granulation tissue, no purulent exudation was present. The patient was then discharged and continued to receive standardized blood sugar control treatment. Follow-up found the ulcer skin recovered 60 days after enrolling in LIDUS therapy (**Figures 2B–F**). Liver and kidney function were reexamined after ultrasound combined with microbubble treatment, and no abnormalities were found. Ankle brachial index was 0.96, slightly decreased. The CPT grade was 7.0, indicating mild hypoesthesia (**Table 1**). After discharge on the 12th day, the following medications were given to control glycemic for the next 15 days, subcutaneous injections of recombinant insulin lyprol (10 IU-8 IU, twice a day), before breakfast and dinner, oral metformin hydrochloride sustained-release tablet (0.5 g, twice a day), oral sitagliptin phosphate tablet(100 mg, once a day). Subsequently, the patient stopped glucose-controlling drugs by himself, and wound dressing was changed daily. To be clear, the timeline of entire treatment process of this case was presented in **Figure 3**.

### Case 2

A 70-year-old male patient diagnosed with type 2 diabetes for more than 10 years had poor glycemic control due to irregular medication. The patient found an ulcer on his right heel without any known injuries eight months ago. After removing the black scab on the surface of the ulcer by himself, the ulcer was getting worse and the patient was subsequently admitted to the Endocrinology Department of our hospital. Since the onset of the ulcer, the patient had complained about a progressively deterioration in mental, physical, appetite and sleep. The patient also had a history of alcohol abuse for more than 30 years (about 500mL strong wine a day).

Physical examination revealed a skin defect about 7.5 × 4.6 cm in size and 4 mm in depth, with black crusts and yellowish exudate, surrounding skin redness and swelling, and pain when walking and pressing the wound (**Figure 1D**). Laboratory examination showed normal fasting blood glucose (PP 5.91 mmol/L), increased HbA1c (8.90%), normal liver function, and Renal insufficiency (blood urea (16.76 mmol/L, serum creatinine 261 umol/L, and serum uric acid 559 umol/L) (**Table 1**).Blood pressure test showed hypertension (160/110 mmHg). MRI examination showed that the ulcer did not involve bone tissue (**Figure 1E**). CDFI showed mild atherosclerosis of lower extremity arteries (**Figure 1F**). Ankle-brachial index was in the normal range, and the CPT grade was 8.37, suggesting moderate hypoesthesia. Based on these evidence, the patient preliminary diagnosed as type 2 diabetes, diabetic foot, hyperlipidemia, hypertension and renal impairment in the outpatient department of endocrinology of our hospital.

Heel ulcer debridement was performed on the patient first (**Figure 2G**). After debridement, the ulcer surface showed no obvious granulation tissue and light red color, and the Achilles tendon was partially necrotic and pale color with partial yellowness. After 7 consecutive days of ultrasound combined with microbubble therapy, the ulcer area did not change significantly (**Figure 2H**). On the 7th day, the granulation tissue grew obviously, and the tendon tissue grew slightly (**Figure 2H**). On the 14th day, the wound contracted slightly and granulation tissue grew with bright red color (**Figure 2I**). On the 30th day, the granulation tissue tended to fill the wound, and a little epidermal tissue grew around it (**Figure 2J**). On the 40th day, the wound contracted and became slightly smaller, granulation tissue protruded from the skin surface, and the tendon tissue was completely repaired with normal color (**Figure 2K**). During the LIDUS therapy, the patient’s local pain and itching gradually increased. Subsequently, the patient was to be treated by skin grafting in the burn department. However, due to the long-term high blood glucose (about 10 mmol/L), the surgeon suggested controlling blood glucose before operating. With no other treatment, after 150 days, the wound was covered with epidermis and the ulcer was basically healed (**Figure 2L**). There was no significant change in liver and kidney function before and after ultrasound treatment, ankle-brachial index increased (ABI=1.32), indicating arterial stiffness, and the CPT grade was 8.37, indicating moderate hypoesthesia (**Table 1**). The patient was treated only in the outpatient department, and glycemic control was simply by oral metformin hydrochloride sustained-release tablets (0.5g, twice a day), which was not effective.

## Discussion

The therapeutic effect in 2 patients was positive and encouraging. The size and depth of the ulcer determined the time to cure, and the ulcer completely healed in 60 to 150 days, and the patients were able to live independently. The safety of the treatment process was also verified. There was no significant difference in liver and kidney function in two patients before and after LIDUS therapy. In addition, there was no ecchymosis on the local body surface, and no thrombosis and other adverse events occurred in local veins and arteries. What is noteworthy is patient 2, whose wound was large and blood sugar fluctuated for a long time and remained high. After careful surgical evaluation, skin grafting was finally abandoned to close the wound. Unexpectedly, after 150 days of slow growth, the skin healed on its own. Both patients were followed up for more than one year and had no recurrence of ulcers. At a recent follow-up visit, Patient 1 said that compared with other methods he had known, receiving our treatment was like undergoing ultrasound examination, which was painless, non-invasive, easy to adhere to, and had definite efficacy, and he was happy to share this treatment with other patients.

To our knowledge, this was the first human trail that used LIDUS combined with microbubbles to treat diabetic foot ulcer. Almost all previous studies on ultrasonic treatment of diabetic foot ulcer were *in vitro* or preclinical (7–9). Until now, the study of low-intensity ultrasonic cavitation in the treatment of human diabetic foot ulcer has not been reported. The only known clinical application of LIDUS plus microbubbles is in the field of tumor therapy. Kotopoulis (10) and Liuzheng (11) have respectively used this method to enhance microcirculation blood supply to pancreatic and breast cancer tumors, and improve the efficacy of chemotherapy.

Blood flow enhancement by LIDUS is the premise of this clinical experiment, which was found in our previous animal studies. When MI was set to 0.3, 5 minutes after ultrasound combined with microbubbles irradiation for VX2 tumor, the tumor blood supply was significantly increased by contrast enhanced ultrasound by a direct visualization method and TIC curve quantitative analysis (12). Similarly, in this study, after the soft tissue around DFU was treated for 20 minutes, its blood perfusion was observed increased by direct visualization, and reached a higher peak intensity (-46 to -42dB) by quantitative analysis in a shorter time and decreased more slowly after treatment. The results showed that vascular resistance of muscle tissue decreased and blood perfusion increased after treatment. *In vivo* studies have reported that low-intensity ultrasound combined with microbubbles irradiating muscle tissue for more than 10 minutes can reverse ischemia up to 24 hours (6).

As a chronic refractory wound, diabetic ulcer healing also involves cell proliferation, angiogenesis and other processes. Several studies have explored the molecular mechanism of low-intensity ultrasound combined with microbubble therapy, namely, the enhanced effect of local blood flow is related to the increased synthesis of local vasodilators Nitric oxide (NO) and prostaglandin (13), while the production of ATPase makes cell proliferation and metabolism more active (6). At the same time, this method can increase vascular endothelial growth factor (VEGF) and other growth factors and promote angiogenesis (14). Hypoxia-inducible factor-α (HIF-α) and the activation of immune pathway are also involved in this process (15, 16). In this case, the wounds of the 2 patients were gradually and slowly healed after short treatment. The timing and molecular mechanism of initiating active wound repair and continuing to heal need to be further studied.

Diagnostic low-intensity ultrasound was selected in this study primarily for the safety of human trials. Due to the strict FDA restrictions (17), the acoustic intensity of diagnostic ultrasound is constrained within an admissible range (0.05-0.5 W/cm^2^) in the form of low energy. As the cavitation nuclei, the microbubbles can reduce the cavitation threshold and enhance the cavitation effect (18, 19). Moreover, the visualization advantage of diagnostic low-intensity ultrasound enables it to observe the whole process of microbubble perfusion and rupture. In the experiment, it was observed that the muscular tissue perfusion microbubbles disappeared after a flash operation. From this, it can be speculated that the existing parameter Settings caused Sonoporation in the treatment, and the physical effects such as shear waves and micro jets may promote the occurrence of healing events. High parameters of MI (0.89 and 0.86), sound power (79%) and Frame (50) were set to improve the duty cycle of treatment pulses, and Flash mode was selected to promote the occurrence of cavitation through breaking microbubbles. The possibility of cavitation induced by commercial equipment was also confirmed by Lindar (6) and Kitoplis (10).

The importance of this study may be reflected in the following aspects: firstly, it was a real world clinical study in humans, and secondly, the therapeutic equipments and microbubbles used are commercial products, which could make this technology promising for clinical promotion and could provide a new non-invasive method and idea for the treatment of diabetic ulcers. The limitations of this study mainly focus on the small sample size, the interpretation of the results needs to be cautious, and more samples need to be accumulated to further verify the treatment effect.

## Data availability statement

The original contributions presented in the study are included in the article/Supplementary Material. Further inquiries can be directed to the corresponding authors.

## Ethics statement

The studies involving human participants were reviewed and approved by the Ethics Committee of The General Hospital of Western Theater Command of PLA. The patients/participants provided their written informed consent to participate in this study. Written informed consent was obtained from the individual(s) for the publication of any potentially identifiable images or data included in this article.

## Author contributions

JT, RW and ZC: conception and design of the work. XZ and LP: data collection. XZ, YC and RW: Image analysis and interpretation, manuscript writing, and critical revision of the article. ZC: approval of the final version of the article.

## Funding

This work received grants from Key Research and Development Program of Science and Technology Department of Sichuan Province (2020YFS0122), and Project of Hospital management of General Hospital of Western Theater Command of PLA (2019ZY11).

## Conflict of interest

The authors declare that the research was conducted in the absence of any commercial or financial relationships that could be construed as a potential conflict of interest.

## Publisher’s note

All claims expressed in this article are solely those of the authors and do not necessarily represent those of their affiliated organizations, or those of the publisher, the editors and the reviewers. Any product that may be evaluated in this article, or claim that may be made by its manufacturer, is not guaranteed or endorsed by the publisher.
